# A computational study of metal–organic frameworks (MOFs) as potential nanostructures to combat SARS-CoV-2

**DOI:** 10.1038/s41598-022-19845-7

**Published:** 2022-09-20

**Authors:** Mohammad Dahri, Mohammad Moein Sadeghi, Samira Sadat Abolmaali

**Affiliations:** 1 Research Center for Pharmaceutical Nanotechnology, Biomedicine Institute, Tabriz University of Medical Sciences, Tabriz, Iran; 2 Center for Nanotechnology in Drug Delivery, School of Pharmacy, Shiraz University of Medical Sciences, Shiraz, Iran; 3 Student Research Committee, School of Pharmacy, Shiraz University of Medical Sciences, Shiraz, Iran; 4 Department of Pharmaceutical Nanotechnology, School of Pharmacy, Shiraz University of Medical Sciences, Shiraz, Iran

**Keywords:** Computational biology and bioinformatics, Computational models, Metals, Virology, SARS-CoV-2, Virus structures

## Abstract

The COVID-19 causative agent, severe acute respiratory syndrome coronavirus 2 (SARS-CoV-2), has a critical surface protein called spike protein (S protein), which is the target of many vaccines and drugs developments. Among non-structural proteins of SARS-CoV-2, main protease (M^pro^) has drawn much attention to itself for designing antiviral drugs since it is very crucial for the virus replication in host cells. In the first part of the present study, the application of metal–organic frameworks (MOFs), one of the developing nanomaterials in the deformation and consequently inhibition of S protein binding to the receptor, angiotensin-converting enzyme 2 (ACE 2), is investigated. In this line, various S protein inhibitors were designed virtually, including ZIF, UIO, and IRMOF that their interactions with S protein and were investigated using molecular dynamics (MD) simulation. The results revealed that ZIF is the best candidate among the investigated MOFs with the least amount of energy interference with S protein. In the second part, the interaction of three-dimensional (3D) MOFs (such as ZIF, IRMOF, and HKUST) with SARS-CoV-2 M^pro^ was investigated. HKUST had the most potent interaction with M^pro^ and showed more promise in deforming this protein's secondary structure among all materials tested. Furthermore, we investigated the interaction of HKUST-OH with M^pro^ to determine the effect of functionalization. The findings of this study could be used in future studies to introduce bioconjugates of MOFs and biological molecules (e.g., antibody or nanobody) or to use MOFs as carriers for antiviral drug delivery.

## Introduction

Since late 2019 (early 2020), COVID-19 has been a viral infection with a high prevalence and mortality rate worldwide^1^. The World Health Organization (WHO) coordinates different governments by declaring the disease pandemic, developing quarantine programs, and launching vaccine mechanisms such as the COVAX program^2^. SARS-CoV-2, as a beta-coronavirus, is well designed in a way that plays a crucial role in its pathogenicity. The SARS-CoV-2 genome is composed of RNA. The virus has four main structural proteins, including spike (S) glycoprotein, nucleocapsid (N) protein, membrane (M) glycoprotein, and a small envelope (E) glycoprotein^3^.

**Figure 1:**
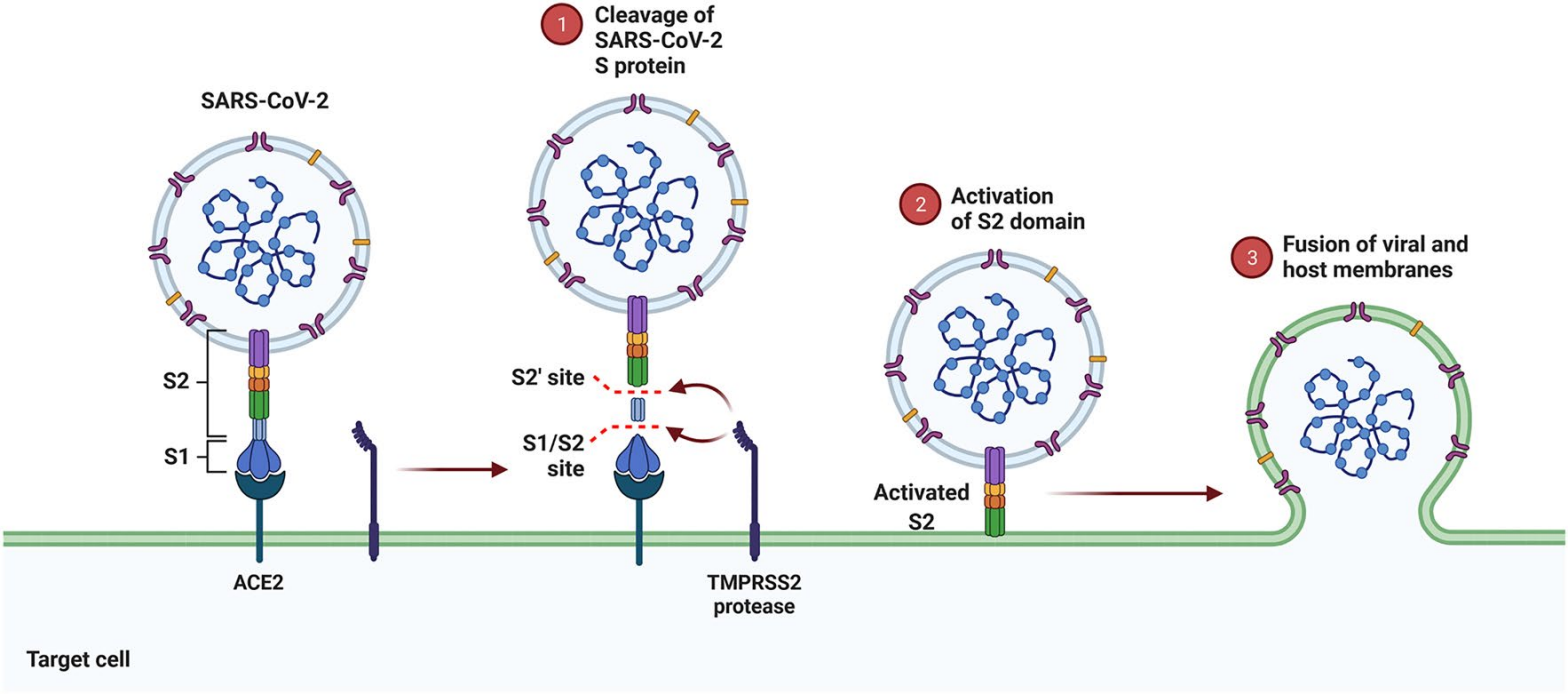
Mechanism of S protein action. (1) The cleavage of S protein to two parts, including S2 and S1 segments. (2) The step of S2 domain activation by binding the S protein to ACE2 receptor. (3) Fusion of viral with the target cell membrane. Reprinted from "Mechanism of SARS-CoV-2 Viral Entry", by BioRender, November 2020, retrieved from https://app.biorender.com/biorender-templates/figures/5e99f5395fd61e0028682c01/t-5f99eb1307ef9a009f1c61e0-mechanism-of-sars-cov-2-viral-entry Copyright 2022 by BioRender.

S protein, which has distinct surface properties, interferes with its specific human cell surface receptors, ACE2. This coronavirus class inhibits ACE2 via the S protein. The S protein is made up of two subunits, S1 and S2. The S1 subunit contains a critical amino acid sequence known as the receptor-binding domain (RBD), which is essential for virus attachment to the host cell ACE2^4,5^. The first step in virus replication is S protein binding to ACE2, which results in respiratory symptoms and high blood pressure. The next step in cell entry is the fusion of the virus membrane with the host cell membrane^6^. The structure of S protein serves as the foundation for developing various vaccines, including those manufactured by Pfizer and Modern^7,8^. In both vaccines, the mRNA translated to the specific part of S protein in the body is loaded into the lipid nanoparticles^9^ (Fig. 1).

In addition to structural proteins, non-structural proteins (NSPs) are important. The SARS-CoV-2 main protease (M^pro^) is one of these NSPs, also known as 3-Chymotrypsin like protease, 3CL^pro^^10^. M^pro^ helps SARS-CoV-2 enter the host body and plays an important role in virus replication. M^pro^ and the papain-like protease (PL^pro^) process SARS-CoV-2-encoded polyproteins^11^. Polyproteins 1a and 1ab (pp1a and pp1ab) are encoded by the virus genome's first and largest open reading frame (ORF1ab). M^pro^ and PL^pro^ cleave pp1a and pp1ab into 16 non-structural proteins (NSP1-16)^12^. One of these 16 NSPs is M^pro^ itself (NSP5). M^pro^ first autoclaves itself then processes the remaining pp1a and pp1ab cleaving points^13^. M^pro^ is pivotal for SARS-CoV-2 survival. This crucial role has received much attention as a drug target for combating COVID-19^14^. Previous SARS-CoV M^pro^ research was used to develop anti-coronaviral drugs. We have insight into developing M^pro^ anti-SARS-CoV-2 medicines due to the high identity of SARS-CoV-2 M^pro^ with SARS-CoV M^pro^^14,15^. In addition to FDA-approved drugs with other indications (e.g. lopinavir/ritonavir^16,17^ and darunavir^18,19^), many new small molecules and peptidomimetics have already been studied in silico and in vitro for SARS-CoV-2 M^pro^ inhibition^20–22^. Cui et al*.*^23^ have reviewed all repurposed drugs, novel molecules, and peptidomimetics that potentially target SARS-CoV-2 M^pro^.

Nanotechnology offers a promising solution for combating viral diseases. The role of nanotechnology in providing efficient therapeutic, diagnostic, and prophylactic platforms for viral infections like COVID-19 is well established^24–26^. MOFs are one of the most applied ones of nanoparticles. MOFs are a type of hybrid material in which organic ligands link an inorganic cluster of metallic ions to form a network^27,28^. The benefits of these crystalline porous nanostructures have been studied in various fields, including environmental and biomedical sciences^29^. Many popular properties of MOFs, such as porosity, high drug loading capacity, the ability to diversely functionalize the surface, and desirable drug release kinetics, make them suitable drug delivery^30^, gene delivery^31^, and magnetic resonance imaging contrast agents^32^. Thanks to their excellent properties, they can act as theranostics^33^ and biosensors for virus detection^21,34–37^.

Herein, we investigated the application of novel 3D MOFs, including ZIF, UIO, IRMOF, HKUST, and HKUST-OH against COVID-19. Using computational tools, we examined the interaction between the considered MOFs with S protein and M^pro^ of SARS-CoV-2 to determine the best sample with the lowest interaction energy. MOFs that strongly interact with SARS-CoV-2 S protein and M^pro^ can be used in many preventive and curative tools like MOF-based face masks to prevent the transmission of the virus, antiviral drugs-loaded MOFs to deliver them to the virus main protease.

## Methods and materials

IRMOF, UIO, ZIF, and HKUST structures were downloaded from https://www.chemtube3d.com/. Also, the charges of the atoms were ‘esp’ charge type. On the other hand, the molecular structure of protease was extracted from (PDB ID: 6LU7, 6W63, 7JQ1, 7JQ4 and 7JQ5) complexes. The structure of these complexes is available at https://www.rcsb.org. In the first part, seven coarse-grained and all-atom simulations and four docking simulations have been performed^38^. On the other hand, three all-atom simulations and three coarse-grained simulations have been performed for spike protein and MOFs. Docking is then performed between ACE2 and spike proteins that have been deformed using MOFs.

In the second part, after preparing the input structural files of the molecules, coarse grain simulations were performed. First, the coarse grain simulation for protease in an aqueous medium was performed. Bulk water was used as representative of aqueous solvent medium. Then, the effect of MOFs on the change of protease structures has been investigated. Three independent coarse-grain simulations of the main protease and MOFs interactions in an aqueous medium were performed to do this. Coarse-grained simulations were performed using the Martini force field. In this regard, the topologies of molecular structures have been created using the Python scripts available on the Martini site (http://cgmartini.nl/index.php/martini). In these simulations, using v-rescale and Parrinello–Rahman algorithms, the simulation box's temperature and pressure were balanced at 1 bar and 300 K, respectively^39^. Then, using LINCS constraints, considering hydrogen bonds (H-bonds) and cut-off radius equal to 2 nm, simulations were performed with steps of 30 fs at 3000 ns. Simulation boxes for these systems were 20 × 20 × 20 nm. For docking simulations, first, the AutoDock Tool-1.5.6 and the gasteiger charge were added to the spike protein PDB file, and polar hydrogen was added to the ACE2 PDB file^40^. Then the files were saved in pdbqt format. Then the docking process of spike protein and ACE2 was performed using the program autodock_vina_1_1_2_linux_x86^41,42^.

## Result and discussion

### Evaluating deformation of spike protein structure by MOFs

**Figure 2:**
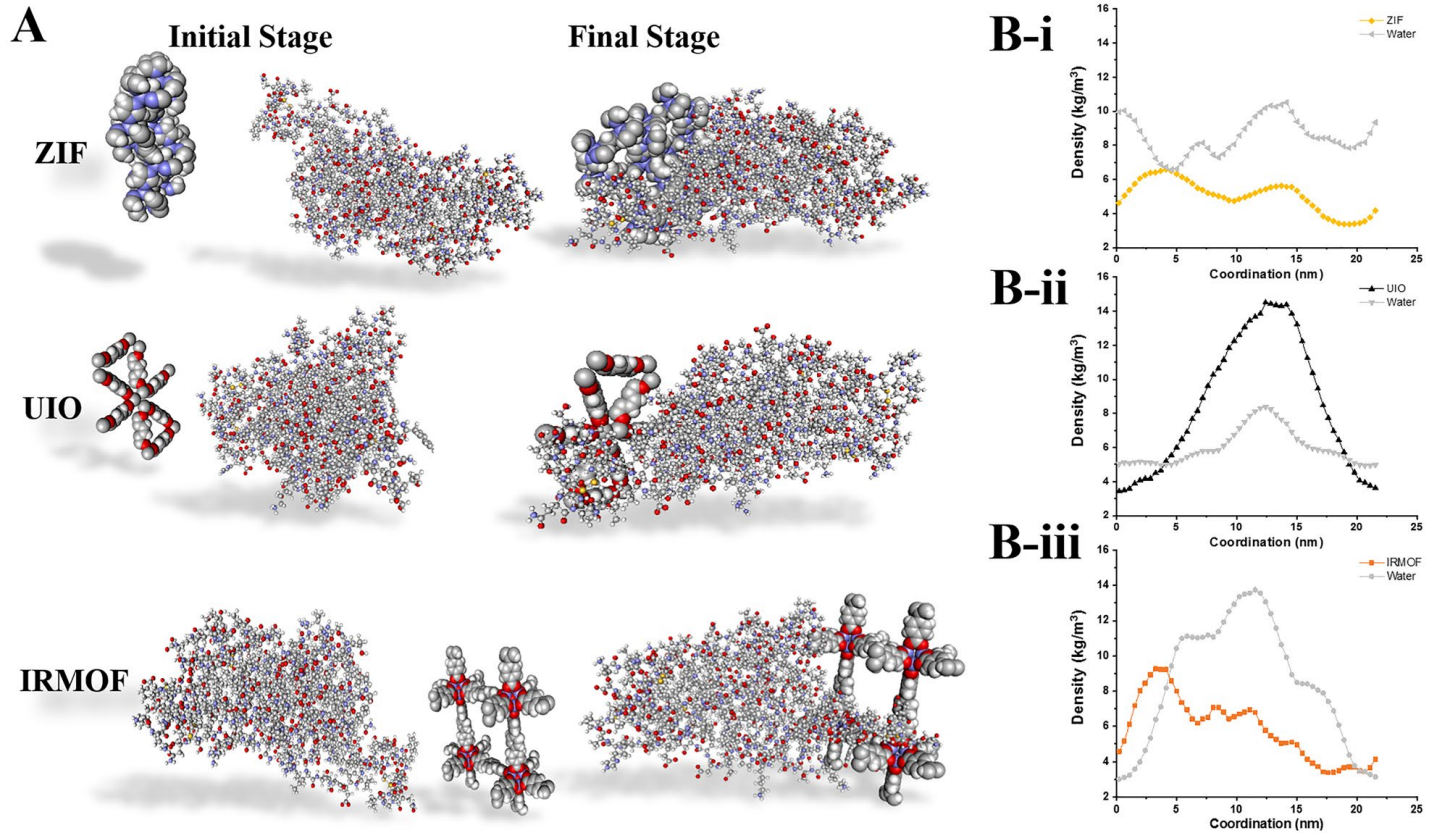
(**A**) Snapshots of the initial and final stages of the interactions between MOF structures with S protein. Water molecules as background are ignored. (**B-i**–**iii**) density distribution of MOF structures and water along with the simulation boxes for ZIF, UIO, and IRMOFs, respectively.

For evaluation, the snapshots of each simulation's initial and final stages are provided in Fig. 2A. As can be seen, MOFs have all been attracted to the surface of S protein. At first glance, it is clear that ZIF covers the most surface area of the S protein, whereas UIO and IRMOF have lower attached surfaces to the S protein. It implies that ZIF interacts with the protein more successfully. In the following sections, all simulations' quantitative evaluations are pursued to understand the interactions better.

Figure 2B represents the MOFs density and water density in the simulation boxes. Atomic distribution density diagram shows the atomic accumulation in the simulation box. The horizontal arrangement of the MOF on the S protein is confirmed by the more even density diagram of ZIF across the box. On the other hand, sharp peaks of UIO and IRMOF across the axes confirm the vertical positioning of the structures with a lower interface with S proteins^43^.

**Figure 3:**
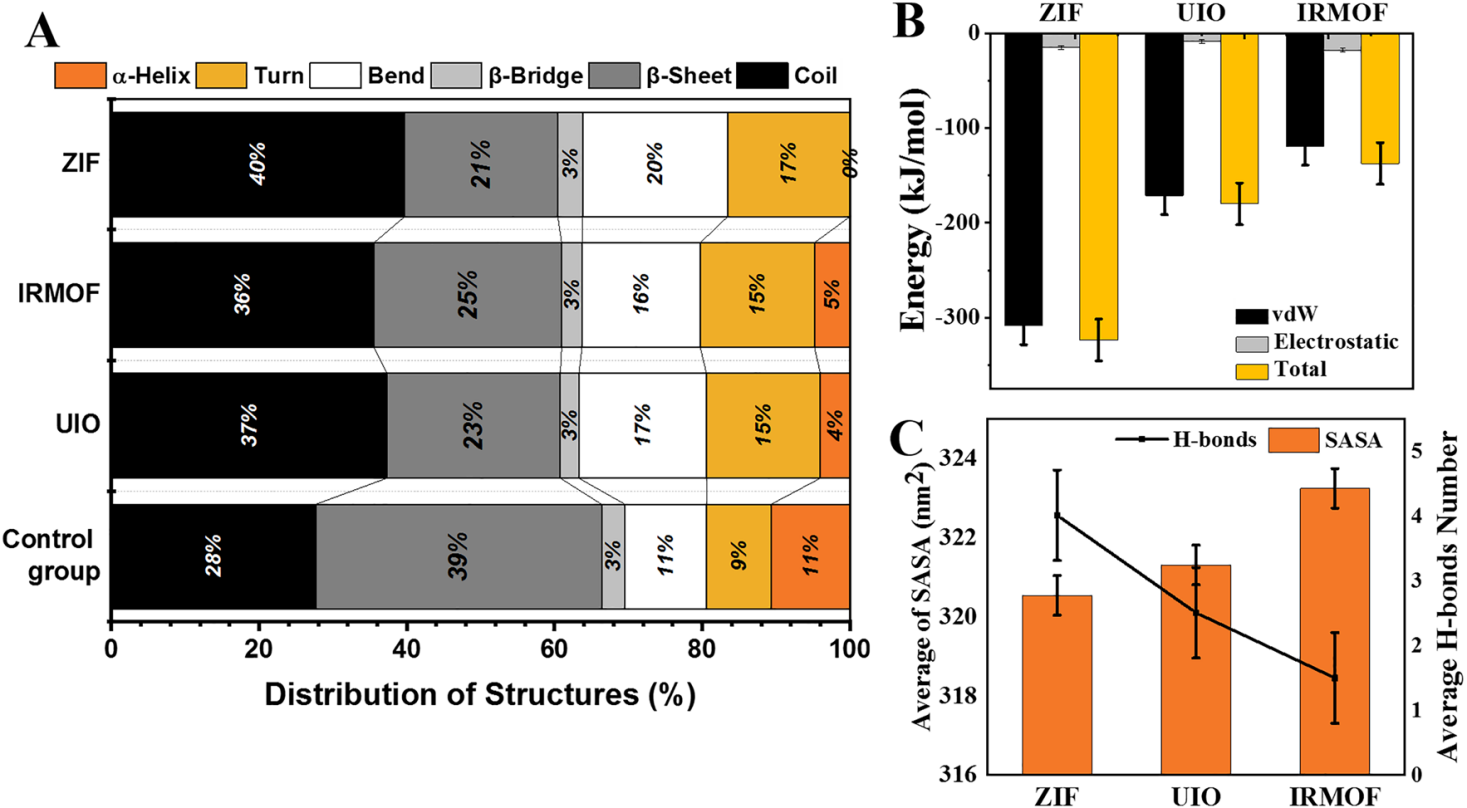
(**A**) Distribution of the spike protein secondary structures after interaction with MOFs. (**B**) Average vdW, the electrostatic and total energy of the spike protein and MOF interactions. (**C**) Average of contact area between SP and aqueous media after interacting with MOFs and average H-bonds between spike protein and MOFs.

Following the interactions with nanomaterials, the structure of S protein can change. To examine the deformation, the distribution of the secondary structures for S proteins after interaction with MOFs has been evaluated (Fig. 3A). Increasing of β-sheets and α-helices intensity and reduction of the coils, bends, and turns indicate the stable structure with more likely interaction with ACE2. As shown in the diagram, pristine S protein with no interaction with MOFs has the most β-sheets in its structure, which can be interpreted as its stable structure and, as a result, its higher capability in interacting with ACE2.S protein after contact with ZIF exhibits the lowest β-sheets (21%) without α-helix in the structure that displays ZIF’s inhibitory effect against S protein function. Moreover, loosely structured proteins (e.g., coil and turn) hit the maximum amount in the secondary structure of S protein after contact with ZIF. Although a few unit cells of MOFs are used in the simulation box, their effects on S protein interaction and deformation can be generally predicted to their nanoparticles which can potentially interact with and deform the S protein^43,44^.

To determine the effect of three various MOFs on S protein–ACE2 complex formation, we evaluated van der Waals (vdW) and electrostatic interactions between S protein and the structures. Since the vdW and electrostatic energies between the molecules are important during structural deformations, these interactions are studied. Figure 3B presents the average energy of vdW and electrostatic interactions between S protein and each MOF. The vdW forces outnumber the electrostatic forces, which is more pronounced in the total energy. ZIF has the highest total binding energy of any spike protein or MOF. The stronger interactions between S protein and ZIF resulted in more deformation in the protein structure by recent MOF. The vdW energy is an accurate indicator of hydrophobic forces. The amount of this force is related to the atomic radius of the MOF. As a result, vdW energy is usually greater at larger atomic radii. This is supported by ZIF's higher negative energy and larger radius. S protein structural changes, on the other hand, are accompanied by an increase in stabilizing structures and a decrease in destabilizing structures as a result of its interaction with ZIF. Low vdW energy and appropriate structural changes are two essential criteria for ZIF to better inhibit this protein.

H-bonds are among the most important intermolecular forces. Increased hydrogen interactions between S protein and MOFs can also alter the structure of S protein and reduce its interactions with ACE2. The average of H-bonds formed between S protein and MOFs is shown in Fig. 3C. The highest and lowest H-bonds with S protein were found in the ZIF and IRMOF structures, respectively. Since these interactions can deform the S protein structure, ZIF is the most effective structure on the S protein secondary structure's instability compared to other investigated MOFs. In this regard, increasing the hydrogen interactions between S protein and MOFs reduces this protein's interaction with aqueous media and reduces its contact area with aqueous media. The average contact area between S protein and MOFs is depicted in Fig. 3C. It reveals that in the presence of ZIF and IRMOF, S Protein had the lowest and highest contact area with aqueous media, respectively. This is due to the high level of hydrogen bonds between ZIF and S protein, which deforms SARS-CoV-2's critical protein structure. The lower the solvent available surface area (SASA), the less nanoparticle solvent available. On the other hand, the greater the number of H-bonds, the stronger the bond between the nanoparticle and the protein. As a result, ZIF has the best interference. Baweja et al*.*^45^ investigated the interaction of a specific protein folding with graphene-based nanoparticles. The result shows an increase in the number of H-bonds and a decrease in the SASA of graphene oxide interaction with protein.

### Evaluation of the effect of S protein deformation on its interaction with ACE2

**Figure 4:**
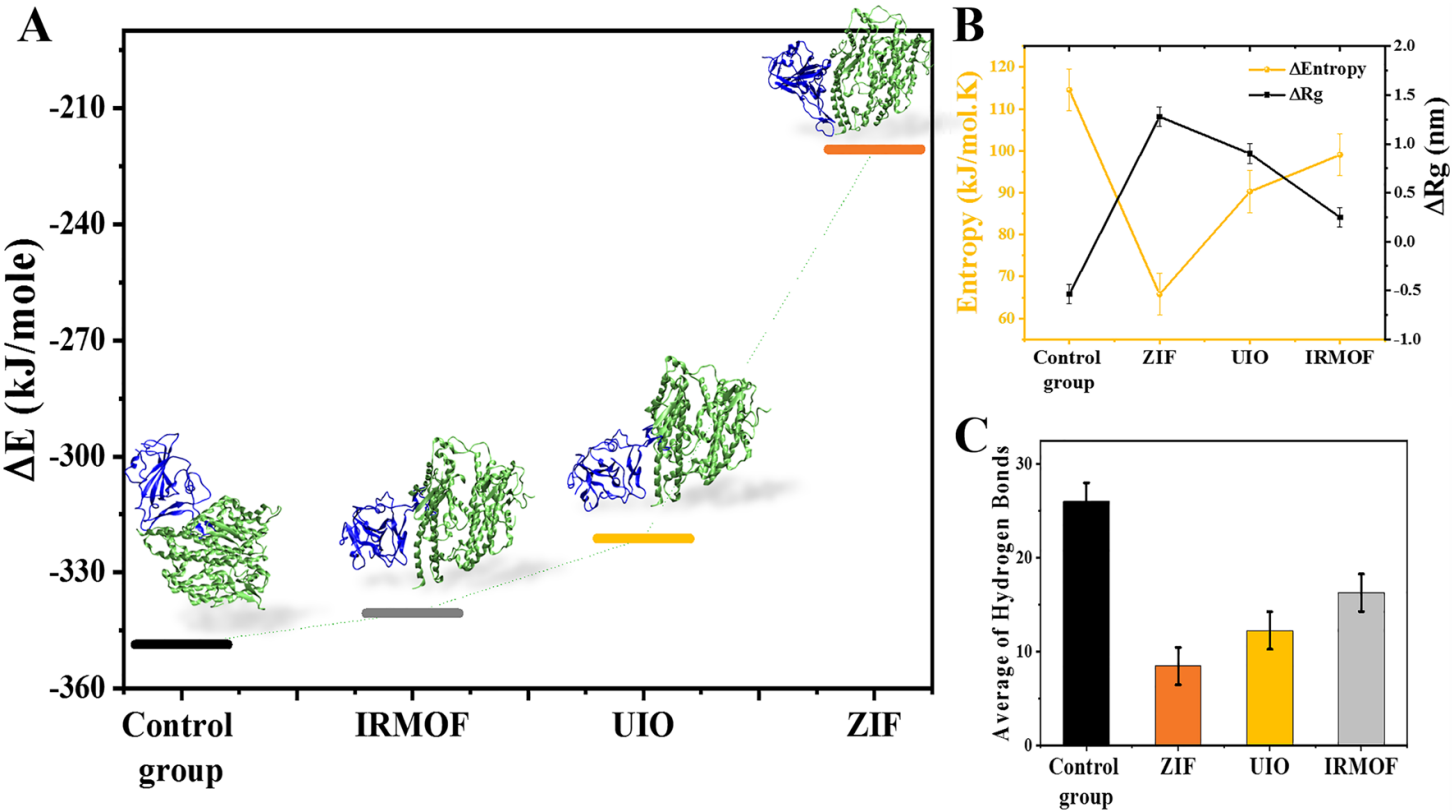
(**A**) Docking interaction energy of (deformed) SPs with ACE2 accompanied with snapshots from simulations. (**B**) Different between final and initial entropy of spike protein–ACE2 interaction as well as Rg of the spike protein. (**C**) Average of hydrogen bonds formed between spike protein and ACE2.

In the previous section, the effect of MOFs on the deformation of the S protein structure was investigated. The current section discusses how S protein deformation affects its interaction with ACE2. Figure 4A reveals the structure of S protein (blue) and ACE2 (green) as well as their interaction energies in the presence of MOFs after docking simulations. The use of deformed S protein structures reduced the energy of the interaction with ACE2 and increased the distance between the S protein and ACE2. Among the considered MOFs, the interaction between ACE2 and ZIF-deformed S protein had the lowest amount of docking energy. Therefore, docking results confirm the previous findings and identify ZIF as the best structure for inducing the S protein structure's deformation. In this regard, the difference in initial and final entropy caused by the interaction of S protein and ACE2 was also investigated and shown in Fig. 4B. The greater the entropy difference, the greater the negative Gibbs free energy, resulting in a more stable S protein interaction with ACE2. Although deformation of S protein by MOFs reduced the entropy difference, the interaction between ACE2 and S protein deformed by ZIF had the lowest entropy difference. This indicates that the S protein–ACE2 complex is unstable due to the deformation of the S protein structure. Entropy analysis, like docking results, shows ZIF as the best structure to deform S protein.

On the other hand, the interaction between S protein and ACE2 causes more compactness of the protein structure. The degree of S protein compaction is revealed by analyzing the gyration radius. The greater the S protein compactness, the smaller the gyration radius. So, in this study, we evaluated the difference in the radius of gyration at the initiation and the end of the simulation as a comparison index of S protein compaction (Fig. 4B). A negative difference in the radius of gyration indicates decreased compactness of the deformed S protein after the interaction with ACE2. The littlest interaction between ACE2 and the S protein deformed by ZIF is observed. This deformed S protein in this simulation had the smallest difference in the radius of gyration. Mousavi et al*.*^46^ investigated the conformational behaviors of chitosan nanoparticles on donepezil and rivastigmine drugs. By varying the ions, the Rg of drugs and polymers was altered. The Rg decreases, and the stability increases as the drug loading increases. In this regard, our findings show that ZIF has the smallest radius and thus the most stable interference. This nanoparticle has the lowest energy and the most stable state in terms of energy. As previously stated, H-bonds are one of the most powerful intermolecular interactions and significantly impact the intermolecular bonds between S protein and ACE2. As a result, studying the H-bonds between S protein and ACE2 is a good indicator of the effects of S protein deformation on its interaction with ACE2. The average of the H-bonds formed between S protein and ACE2 is shown in Fig. 4C. According to the findings, deformation of S protein by MOFs reduced H-bonds, indicating the effectiveness of S protein deformation in reducing the interaction with ACE2. Because ZIF was more effective at reducing hydrogen interactions, it is the best structure for deforming S protein.

### Main protease structural variation after interaction with 3D MOFs

**Figure 5:**
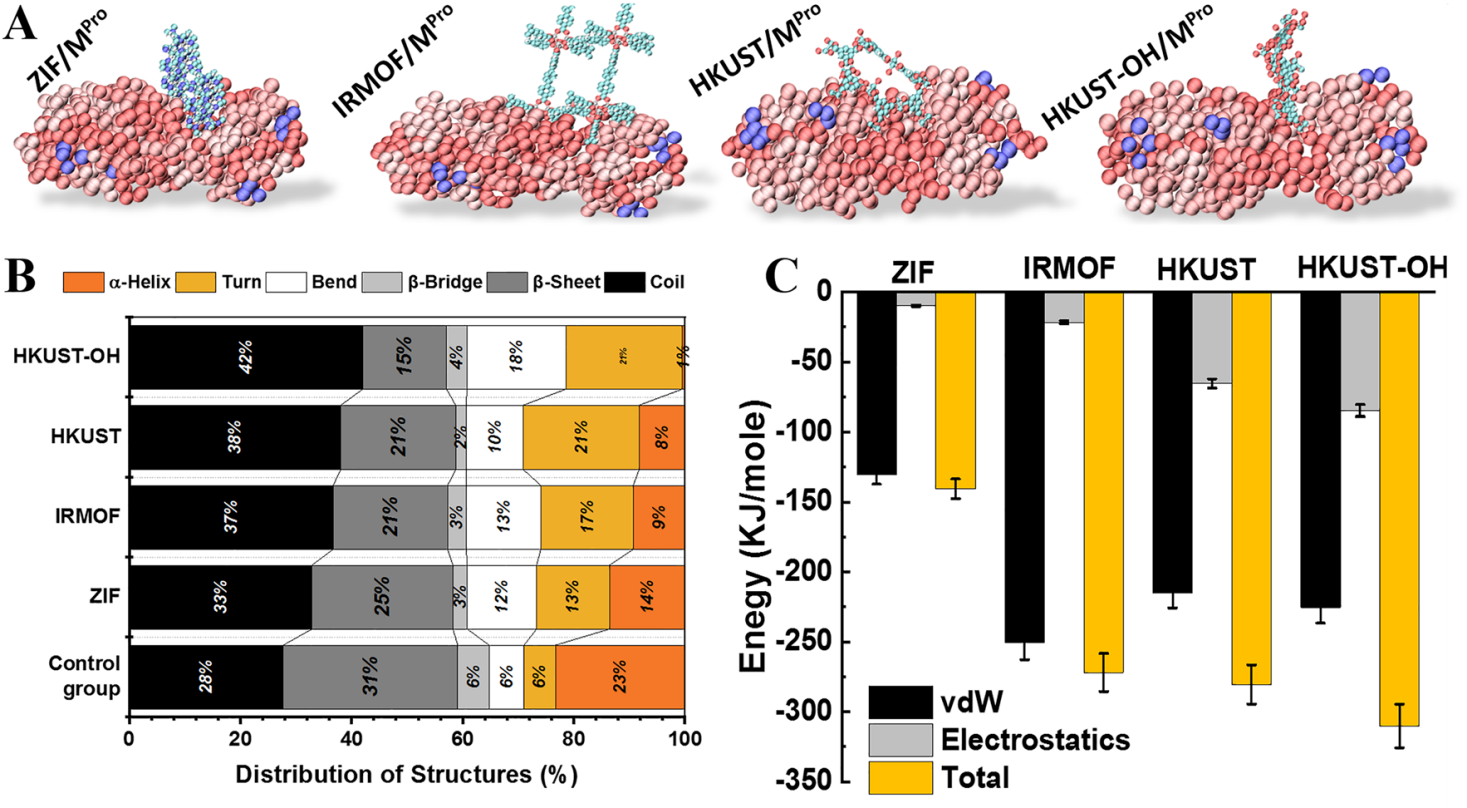
Evaluation of the MOFs’ impact on M^pro^ structure: (**A**) Snapshots of the simulation of COVID-19 M^pro^ with MOF nanostructures, (**B**) distribution of secondary structure of MOFs and control group, (**C**) energy of interaction.

The importance of SARS-CoV-2 M^pro^ in the virus replication cycle was explained. The effects of 3D structures, including ZIF, IRMOF, and HKUST, on various secondary structures of the enzyme, were investigated, and snapshots from the last stage of simulations are provided in Fig. 5A. The amount of each secondary structure of the enzyme, including β-sheets, helices, β-bridges, turns, bends, and coils, changed after interaction with all investigated MOFs. The distribution of the secondary structures of M^pro^ (Fig. 5B) demonstrates that the percentage of the coil, turn, and bend structures of the enzyme increased after interaction with the mentioned MOFs. HKUST had the greatest increase in coil, turn, and bend structures. Thus, all 3D MOFs weakened the enzyme structure stability compared to the control group (pure enzyme), while HKUST induced instability more than other 3D materials. As is well-known, surface engineering and modification can improve the properties and performance of nanomaterials. In this regard, we investigated the effect of the hydroxyl group on the HKUST as the MOF with the best performance in the considered group. As expected, functionalized HKUST (HKUST-OH) yielded the highest degree of instability, even more than pristine HKUST. As is obvious, the structures that cause the most changes in the enzyme structure, from most to least, are as follows: HKUST-OH, HKUST, IRMOF, and ZIF. In a similar study, Jin et al*.*^47^ investigated the effect of graphene oxide nanosheets on the secondary structure of β-amyloid using DPPS analysis. Exposure to graphene oxide nanosheets increased the percentage of coil structures and decreased the percentage of β-sheets of β-amyloid. Simulation results show that nanomaterials destabilize the protein structure, which is consistent with their research.

To gain deep insight into the impact of nanomaterials on the SARS-CoV-2 M^pro^ using g_mmpbsa software^48^, the interactions are analyzed from an energetic point of view, including vdW and electrostatics as well as total energy (Fig. 5C). Negative energy values indicate stable interaction between the corresponding nanomaterial and the unaffected enzyme (as the control group). Surface modification of HKUST by adding hydroxyl groups led to boosted vdW and electrostatic interactions. It can be attributed to the enlarged HKUST structure due to the presence of functional groups that consequently strengthen vdW attractions. Furthermore, because of the presence of negative -OH groups, the electrostatic energy was amplified, causing the protein to be more strongly adsorbed toward HKUST-OH. On the other hand, the vdW interaction of M^pro^ with IRMOF is significantly stronger than with other MOFs. It can be explained by the presence of iron in this framework, which increases the structure's vdW radius and thus amplifies this attraction. However, in the case of HKUST-OH, the electrostatic interactions also add up to the total interactions and cause stronger attractions of HKUST-OH with the protein. Altogether, surface modification of the HKUST modifies both vdW and electrostatic adsorptions, i.e., surface engineering plays a critical role in the design of nanomaterials against COVID-19.

**Figure 6:**
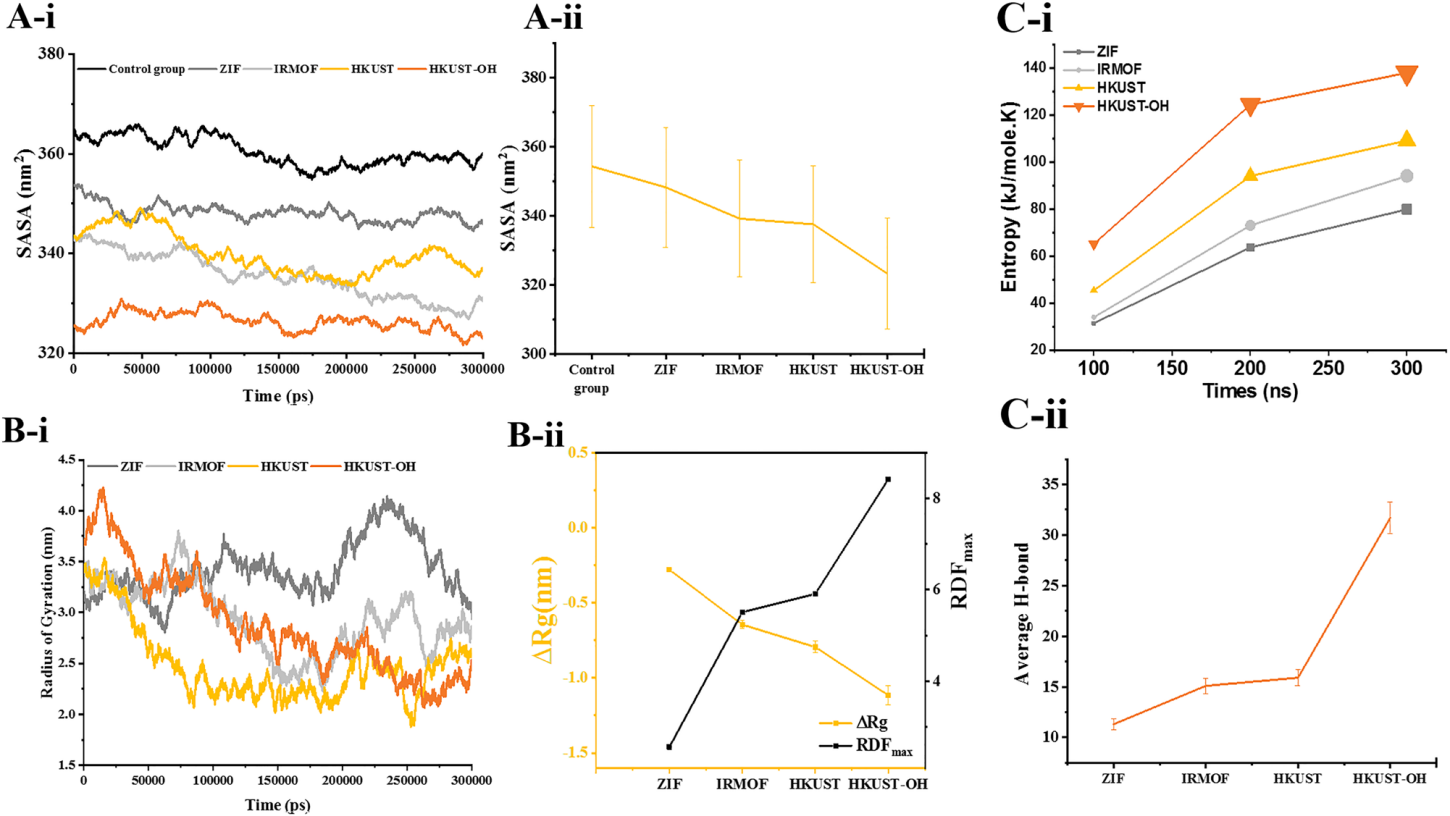
(**A-i**,**ii**) SASA of deformed M^pro^ with MOFs together with the control group for comparison purposes in the course of simulation and average, respectively. (**B-i**) Variation of Rg over the simulation time of MOFs’ interaction with M^pro^. (**B-ii**) The difference between final and initial Rg (ΔRg) of the M^pro^ in the interaction with MOFs to get more insight on the compactness of each case together with maximum radial distribution function (RDF). (**C-i**) Entropy of M^pro^-MOF interaction over the simulation time. (**C-ii**) Average numbers of H-bonds between MOFs and M^pro^.

SASA (during the simulation and average values) for the SARS-CoV-2 M^pro^ with or without (control group) nanomaterials is shown in Fig. 6A. As can be seen, the SASA amount is lowest for M^pro^ in the presence of HKUST-OH, indicating a shorter distance between the nanomaterial and the enzyme. In other words, M^pro^ is mostly in contact with HKUST-OH rather than being exposed to solvent. Therefore, the interaction between HKUST-OH and the enzyme is stronger among its peers. To compare the relative exposure of M^pro^ in the presence of nanomaterials, the average SASA for all cases is provided in Fig. 6A-ii. Apparently, the use of pristine HKUST and even surface engineered HKUST-OH reduce SASA of SARS-CoV-2 M^pro^ i.e., accessible surface area of the enzyme and its functionality is reduced.

As variation in Rg decreases, the systems become denser and more petite. The greater the difference, the smaller and denser in similar systems (Fig. 6B-i). As it can be seen in Fig. 6B-ii, the difference between the final and initial Rg is lowest for HKUST-OH, indicating the best interference for these MOFs. Rg analysis results confirm the previous SASA results. Chen et al*.*^49^ used molecular dynamics simulation to demonstrate that the addition of graphene oxide nanosheets reduces the Rg of beta-amyloid. Our findings confirm the findings from SASA analysis on the reduced surface area and functionality of SARS-CoV-2 M^pro^, which are consistent with their findings. The radial distribution functions (RDF) parameter that can be obtained from different methods is used to investigate the molecular aggregation at a specific simulation box location. Compared to molecular aggregation between several systems, the higher the maximization of this factor, the greater the system's molecular aggregation. According to Fig. 6B-ii, the highest values of RDF are detected with HKUST-OH, HKUST, IRMOF, and ZIF, respectively. Therefore, the complex of HKUST-OH and M^pro^ has the best molecular aggregation and accumulation. In another study, Kamel et al.^50^ investigated the effect of different amino acid adsorption on the functional and non-functional nanoparticles. In contrast to our present work, they obtained less RDF for interference between amino acids and functional nanoparticles than non-functional nanoparticles. These differences in graphs can be related to the intrinsic properties of materials and groups.

The impact of nanomaterials on the SARS-CoV-2 M^pro^ is evaluated through entropy calculations. In this regard, the entropy of each simulation is computed at different stages of interaction (Fig. 6C-i). For all cases, the entropy of the system increases by progress in time. However, for the simulations of SARS-CoV-2 M^pro^ in HKUST’s presence, the increase is more accentuated. Entropy increases with surface modification of HKUST with hydroxyl groups, indicating that the enzyme's functionality has been lost due to interactions with MOF molecules.

The presence of hydrogen atoms attached to electronegative atoms (such as fluorine, oxygen, and nitrogen) is important to form H-bonds. The amount of H-bonds between the enzyme and the nanomaterials indicates the bonding as well as the strength of the interference between them. This analysis is a vital and exciting criterion for predicting the interaction between the enzyme and nanomaterials. As the distance between the MOFs and the enzyme decreases, the number of H-bonds increases, i.e., the H-bonds number surges with the progress in the simulation. Figure 6C-ii represents the average H-bonds for each case. A correlation between H-bond number and SASA values seems necessary. With a decrease in the distance between the nanomaterial and the enzyme, the exposed surface area toward water molecules increases. In other words, water molecules between protein and nanomaterial are squeezed out; hence, protein and nanomaterial absorb each other.

Consequently, increases in the H-bonds are observed as a result. Comparing Fig. 6A and C-ii depicts the hypothesized correlation that increased H-bond number results in the decreased available surface area toward solvent molecules. The H-bond number increases with the addition of the hydroxyl functional group that provides more positions for H-bonding with water molecules. By functionalizing the HKUST MOFs with hydroxyl groups, the average number of hydrogen bonds increased from 16 to about 34. Hydrogens attached to the electronegative oxygen atoms provide the condition for forming H-bonds between hydroxy HKUST and M^pro^. The formation of these bands makes this interference stronger.

Table 1 shows the average of root-mean-square deviation (RMSD) and root-mean-square fluctuation (RMSF) for the control group and MOFs during the time. Low values of RMSF and RMSD indicate more stability and balance in the simulation system. The addition of the hydroxyl group has stabilized HKUST MOFs. In addition to having the minimum amount of RMSD, it also has the minimum amount of RMSF. As shown in Fig. 6, the best interaction between the enzyme and the pristine MOFs isf formed by HKUST-OH. The hydroxyl groups promote the MOFs’ interaction with the enzyme.

**Table 1:** The average amount of RMSD and RMSF for the control group and MOFs.

Structure	Average of RMSD (nm)	Average of RMSF (nm)
Control group	5.16	8.34
ZIF	4.85	6.17
IRMOF	4.31	3.81
HKUST	4.11	3.74
HKUST-OH	3.35	2.91

These findings can be used as a preliminary study for further investigations on MOFs for disrupting COVID-19 replication cycle in which other aspects of MOFs applications should be considered. Although there are some concerns regarding some specific MOFs stability in water and their ability for cell penetration, these parameters can be optimized by introduction of functional groups to the MOF structure and controlling their size^51–53^.

## Conclusion

In this study, various materials with the potential to deactivate the S protein of SARS-CoV-2 were considered to prevent SARS-CoV-2 from entering and infecting human cells. For this purpose, we used three MOFs (ZIF, UIO, and IRMOF) to alter the structure of the S protein and prevent it from interacting with ACE2. The effect of MOFs on spike protein deformation was computed using MD and docking simulations. To do this, DSSP was used to investigate changes in the secondary structure of the spike protein caused by MOF, the intensity of the spike protein and MOF interaction, the contact area of this protein with aqueous media, and hydrogen bonds formed between the spike protein and ACE2. Although all MOFs deformed the S protein, the interaction energy and H-bonds between ZIF and S protein were higher, resulting in the greatest change in the protein's secondary structure. The interaction of deformed S proteins with ACE2 was then investigated. Despite the fact that the results showed a reduction in this interaction, the interaction of ACE2 and S protein deformed by ZIF reached its lowest value with the lowest docking energy, entropy difference, H-bonds, and spike protein compaction. All of these findings point to ZIF as a strong structure capable of deforming the S protein. The MOFs presented in this study deform the S protein structure and prevent it from binding to ACE2, thereby preventing SARS-CoV-2 replication in the body. If the preferred MOF is used ex vivo (e.g. in face masks, air filters, surface coatings, etc.) it has the potential to alter S protein structure and disable it from future interactions with ACE2 after the virus entry to body^54^. Also, if the preferred MOF is administered in vivo for potential applications (e.g. as carriers for antiviral drug delivery, part of bioconjugate system, biosensors, etc.) it can potentially capture S protein and compete with ACE2 for attachment to S protein.

Another way to stop SARS-CoV-2 replication in the body is to prevent it from replicating in the body after infection. Another important drug target in preventing the virus-cell cycle progression is the SARS-CoV-2 main protease (Mpro). The interaction of this enzyme with four MOFs (ZIF, IRMOF, HKUST, and HKUST-OH) was investigated using computational methods in the current study. According to the findings, hydroxy HKUST MOFs form the best interference because this MOF fundamentally alters the secondary structures of M^pro^, resulting in decreased stability. As a result, it prevents the disease from spreading further in the body. Our findings may pave the way for developing new COVID-19 preventive, diagnostic, and curative methods, such as MOF-based air filters, biosensor probes, drug cargos, bioconjugates, and so on.

## Data Availability

The datasets generated and analyzed during the current study are available in the The Protein Data Bank repository, https://www.rcsb.org/ and also in the chemtub3d database, https://www.chemtube3d.com/.

## Abbreviations

CCR2: CC chemokine receptor 2
CCL2: CC chemokine ligand 2
CCR5: CC chemokine receptor 5
TLC: Thin-layer chromatography
